# On the taxonomic position of the enigmatic genus *Tonkinodentus* Schileyko, 1992 (Chilopoda, Scolopendromorpha): the first molecular data

**DOI:** 10.3897/zookeys.840.33635

**Published:** 2019-04-17

**Authors:** Arkady A. Schileyko, Evgeniya N. Solovyeva

**Affiliations:** 1 Zoological Museum of the Moscow Lomonosov State University, Bolshaya Nikitskaja Str. 6, Moscow, 103009, Russia Zoological Museum of Moscow Lomonosov State University Moscow Russia

**Keywords:** Extended redescription, molecular analysis, Scolopendridae, taxonomic position, *
Tonkinodentus
*, 18S rRNA, 28S rRNA

## Abstract

The taxonomic position of the monotypic Vietnamese genus *Tonkinodentus* Schileyko, 1992 (for *T.lestes* Schileyko, 1992) has been considered in the light of the first obtained molecular data. Both molecular (28S rRNA) and morphological data support the position of this extraordinary eye-less genus within the family Scolopendridae Leach, 1814, a sighted clade, and thus suggests the polyphyly of blind scolopendromorphs. The species diagnosis has been amended and color images of *T.lestes* provided for the first time.

## Introduction

**Figures 1–4.**
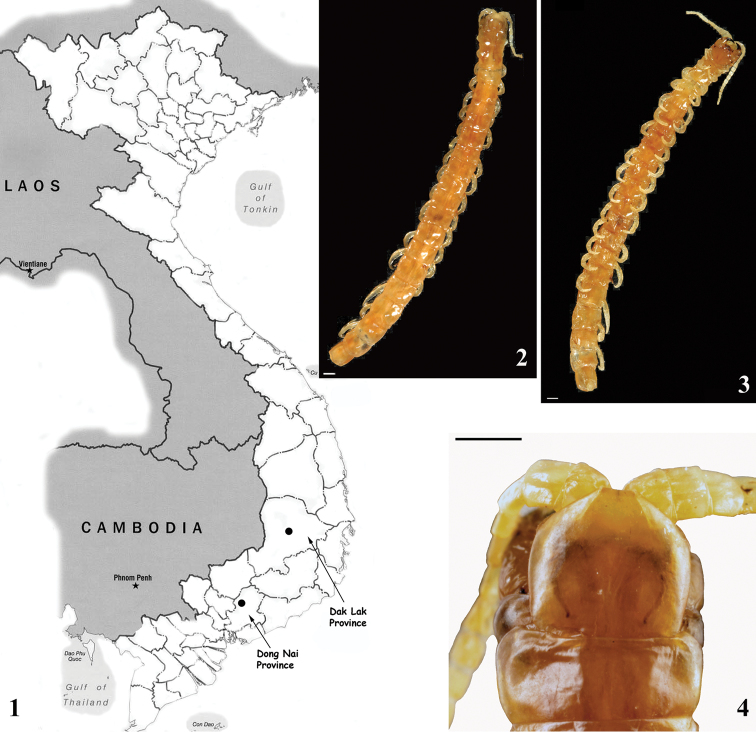
**1** Map of Vietnam showing the places of collection (black circles) of the holotype (Dak Lak Province) and the second specimen (Dong Nai Province) of *Tonkinodentuslestes* Schileyko, 1992; *Tonkinodentuslestes* Schileyko, 1992, holotype (Rc 6358) **2** general view, dorsally **3** general view, ventrally **4** head plate and LBS 1, dorsal view.

**Figures 5–9.**
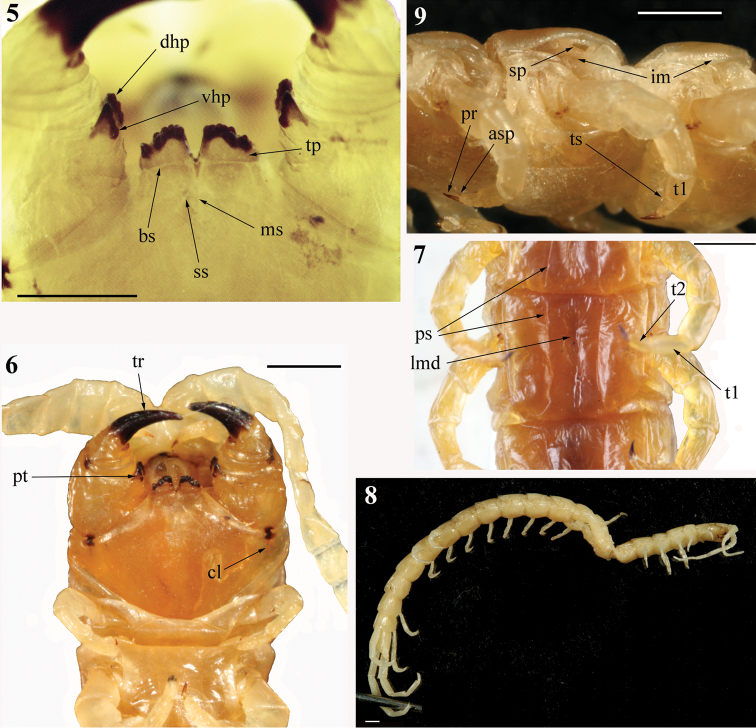
*Tonkinodentuslestes* Schileyko, 1992; Holotype (Rc 6358) **5** anterior margin of forcipular coxosternite, ventral view **6** head, forcipular segment and LBS 1–2, ventral view **7**LBS 12, ventral view; non-type (Rc 6555) **8** general view, laterally; Holotype (Rc 6358) **9** left side of LBS 2–4, lateral view; (**asp**) – accessory spines, (**bs**) – basal sutures of tooth-plates, (**cl**) – chitin-line, (**dhp**) – dorsal half of process of trochanteroprefemur, (**im**) – pleural intersclerite membrane, (**lmd**) – longitudinal median depression, (**ms**) – medial suture, (**pr**) – leg pretarsus, (**ps**) – paramedian suture, (**pt**) – process of trochanteroprefemur, (**sp**) – slit-like spiracle of LBS 3, (**ss**) – short caudo-lateral suture, (**tp**) – tooth-plate, (**tr**) – tarsungula, (**ts**) – leg tarsal spur, (**t1**) – leg tarsus 1, (**t2**) – leg tarsus 2, (**vhp**) – ventral half of process of trochanteroprefemur.

The monotypic genus *Tonkinodentus* Schileyko, 1992, based on *T.lestes* Schileyko, 1992, was described from Vietnam by a single adult specimen lacking the ultimate pair of legs. This enigmatic taxon, originally collected from Boun Ma Thuout in Dak Lak Province (Fig. 1), is extraordinary in lacking eyes (Figs 2, 4) but otherwise scolopendrid-like in all other aspects. According to Schileyko (1992), the presence of forcipular tooth-plates and a trochanteroprefemoral process (Figs 3, 5, 6) and the absence of a sternal transverse suture (Fig. 7) place *Tonkinodentus* in Theatopsinae Verhoeff, 1906 (in the sense of Schileyko 1992). Shelley (1997) synonymised Theatopsinae with Plutoniuminae (= Plutoniumidae) Bollman, 1893 and also removed *Tonkinodentus* from this subfamily.

**Figures 10–14.**
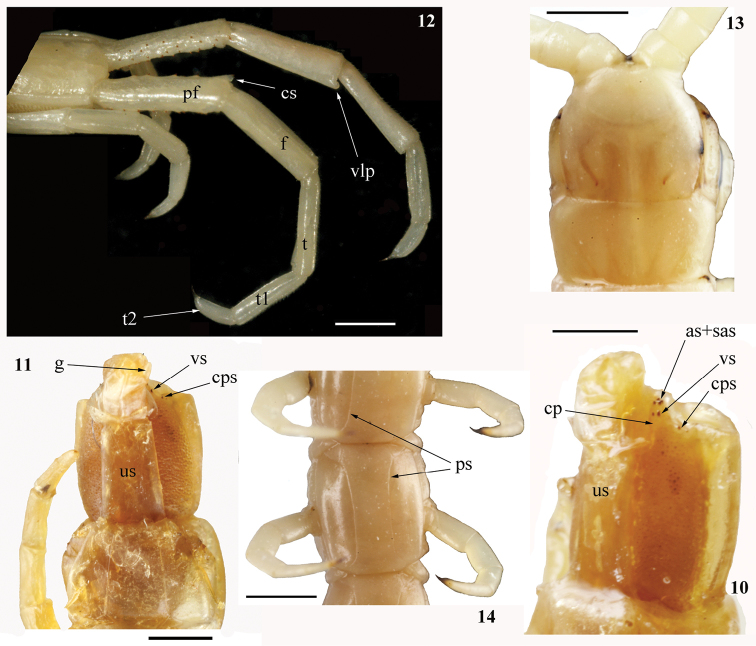
*Tonkinodentuslestes* Schileyko, 1992; Holotype (Rc 6358) **10**LBS 20–21, ventro-lateral view **11**LBS 20–21, ventral view; non-type (Rc 6555) **12**LBS 21 and ultimate legs, dorso-lateral view **13** head plate and LBS 1, dorsal view **14**LBS 13, ventral view; (**as**) – apical spine(s) of coxopleural process, (**cp**) – coxopleural process, (**cps**) – coxopleural posterior spine, (**cs**) – corner spine of ultimate prefemur, (**f**) – femur, (**g**) – gonopod, (**pf**) – prefemur, (**ps**) – paramedian sutures, (**sas**) – subapical spine(s) of coxopleural process, (**t**) – tibia, (**t1**) – tarsus 1, (**t2**) – tarsus 2, (**us**) – ultimate sternite, (**vlp**) – distal ventro-lateral process, (**vs**) – ventral spine(s) of coxopleural process.

In 1994 another (complete) subadult specimen of *T.lestes* (Fig. 8) was found in Dong Nai Province (Fig. 1), and the species was redescribed by Schileyko (2007). As a result, it turned out that *Tonkinodentus* has (as the overwhelming majority of the subfamily Scolopendrinae Leach, 1814) paired sternal longitudinal sutures (Fig. 7), slit-like spiracles (Fig. 9) covered by “flap” (a synapomorphy that is unique for Scolopendrinae), a well-developed and spinulated coxopleural process (Figs 10, 11), and regular (“common” sensu Schileyko 2009) ultimate legs (Figs 8, 12). Thus, *T.lestes* is morphologically much closer to the sighted Scolopendrinae rather than to the blind Plutoniumidae. The subsequent cladistic analyses of Edgecombe and Giribet (2004), Vahtera et al. (2012a), and others (see below), which were based on both molecular and morphologic data, proposed the monophyly of the blind scolopendromorphs (i.e. Cryptopidae sensu Attems, 1930); however, they did not contain data on *Tonkinodentus*.

Taking into consideration all these facts, Schileyko (2007) supposed *Tonkinodentus* to be the first blind representative of Scolopendridae. Schileyko (2007: 71) also wrote that a discussion concerning taxonomic position of this genus will be published elsewhere and left *Tonkinodentus* unassigned to any subfamily. Thus, the aim of this paper is to specify a taxonomic position of this enigmatic genus using the first molecular data as presented below.

## Material and methods

All the studied material is deposited in the Zoological Museum of Moscow Lomonosov State University (ZMMU). The work was carried out based on the two specimens of *Tonkinodentuslestes* (Rc 6358, holotype; Rc 6555, non-type). Abbreviations used are LBS = leg-bearing segment(s), col. = collector. Specimens were examined both wet and dry under various angles of direct illumination; the photos were taken using a Canon EF-S 60 macro lens mm mounted on Canon EOS 300 camera and DeltaPix Invenio-8DII digital camera. Lewis et al. (2005) and Bonato et al. (2010) were followed for standard terminology of centipede morphology.

A tissue sample of *T.lestes* was taken from the 75% ethanol preserved specimen (voucher number Rc 6555 in ZMMU, collected in 1994). To avoid contamination, extraction and amplification of the DNA were carried out in the ZMMU Laboratory of Historical DNA. This laboratory was specially designed for work with samples from museum specimens, which potentially have their DNA degraded. No previous work on fresh tissues had been performed in this laboratory (Kruskop et al. 2018; Lebedev et al. 2018). DNA was extracted twice; for the first time, it was extracted and purified using the QIAamp DNA MiniKit (Qiagen), which included an overnight lysis step at 56 °C and longer incubation with EB-buffer (5 min) at the purification step. For the second time, DNA was extracted using a non-destructive method (Gilbert et al. 2007) with the following modifications: incubation at 55 °C was performed for 8 h, DNA purification was done with Qiagen PCR purification kit.

We amplified a fragment of the 28S rRNA nuclear gene. The DNA was highly degraded, so short fragments (100–200 bp) were obtained using the combination of internal primers designed for this study (Appendix 1, 2). Primer pairs were developed manually using Bioedit (Hall 1999) and an alignment of candidate centipede sequences from GenBank. First DNA extraction was successfully amplified with 28S-endF/28S-endR primer pairs, but another pair of primers (startF_2 and IntR_2) worked only on the second DNA extraction.

The PCR program for amplification of short fragments included an initial denaturation at 95 °C for 3 min, 45 cycles of 95 °C for 30 s, annealing temperature (see Appendix 1) for 30 s and 72 °C for 30 s, and a final extension of 72 °C for 6 min. All stages of the extraction process included a negative control run in parallel. PCR products were visualized on a 1% agarose gel. PCR product was sequenced via Evrogen on ABI PRISM 3500xl sequencer. All sequences were deposited in GenBank under the following accession number MK517656.

Additional sequences of 28S rRNA and 18S rRNA of various scolopendromorphs (including the members of Scolopendrinae, i.e. potential close relatives of *T.lestes*) were downloaded from GenBank (see Appendix 3). *Craterostigmustasmanianus* Pocock, 1902, a member of Craterostigmomorpha, was used as an outgroup. We did not increase the length and variability of our alignment by adding mitochondrial DNA data available for this set of taxa (excluding *T.lestes*) in GenBank because the Chilopoda mtDNA sequences are very variable, much more than their nuDNA ones and there is a high possibility of saturation of mtDNA while comparing distant taxa and because the cases of mitochonrial introgression are rather common. We hold to an opinion that in such situations combining both nuDNA and mtDNA data in one alignment can lead to errors and is better avoided, especially when, as in our case, the DNA fragment of the target specimen is short and represents only one type of DNA markers (either nuDNA, or mtDNA).

Sequences of *T.lestes* were checked and put in contig using Seqman 5.06 (Burland 1999). Than contig and GenBank sequences were aligned with Geneious 11.1.5 (http://www.geneious.com) using Geneious Alignment. Subsequently, the alignment was checked and manually revised if necessary using BioEdit Sequence Alignment Editor v. 7.1.3.0 (Hall 1999). Two alignments were prepared for the following phylogenetic analysis: sequences of 28S only and concatenated alignment of 28S + 18S. We did not cut the sequences of the other taxa to match the length of the *T.lestes* fragment, the length of 28S alignment was 1743 b.p. and 1913 b.p. for 18S alignment. 18S sequences were added to improve the resolution of the resulting trees. Genetic distances were calculated using MEGA 6.1 (Tamura et al. 2013).

The optimum partitioning schemes were identified with PartitionFinder (Lanfear et al. 2012) using the greedy search algorithm under the AIC criterion: GTR + I + G for 28S and SIM + I + G for 18S. Phylogenetic trees were reconstructed under Bayesian criteria (BI) and the maximum likelihood (ML). Bayesian inference (BI) was performed in MrBayes v. 3.1.2 (Ronquist and Huelsenbeck 2003) with two simultaneous runs, each with four chains, for 8 million generations for 28S and 12 million generations for 28S + 18S. We checked the convergence of the runs and that the effective sample sizes (ESS) were all above 200 by exploring the likelihood plots using TRACER v. 1.5 (Rambaut and Drummond 2007). The initial 10% of trees were discarded as burn-in. Confidence in tree topology was assessed by posterior probability (PP) (Huelsenbeck and Ronquist 2001).

The ML trees were generated in IQ tree (Nguyen et al. 2015) using ultrafast bootstrap = 10000 (UFBoot, Minh et al. 2013). A model for the 28S (GTR+F+I+G4) alignment was selected using ModelFinder (Kalyaanamoorthy et al. 2017), and a partitioning model for the 28S + 18S alignment were calculated with IQtree (Chernomor et al. 2016): GTR+F+I+G4 for 28S and TNe+I+G4 for 18S.

## Results

### 1. Amended diagnosis and redescription of *Tonkinodentuslestes* Schileyko, 1992

Schileyko (2007) redescribed *T.lestes* in detail, but the black-and-white photographs are far from satisfactory. Below we present a new diagnosis and description of this species accompanied by color photographs.

### Family Scolopendridae Leach, 1814

#### Subfamily Scolopendrinae Leach, 1814

##### Genus Tonkinodentus

Taxon classificationAnimaliaScolopendromorphaCryptopidae

Schileyko, 1992

###### Type species.

*Tonkinodentuslestes* Schileyko, 1992 (by monotypy).

###### Range.

Central Vietnam, Dak Lak (Darlak) Province; South Vietnam, Dong Nai Province.

##### Tonkinodentus lestes

Taxon classificationAnimaliaScolopendromorphaCryptopidae

Schileyko, 1992

Figures 2–4 , 5–9 , 10–14 , 15–19

Tonkinodentus lestes Schileyko 1992: 13.
Tonkinodentus lestes : Schileyko 1995: 74.
Tonkinodentus lestes : Schileyko 2007: 83.

###### Locus typicus.

Central Vietnam, Dak Lak (Darlak) Province, environs of Boun Ma Thuot.

###### Material.

Dak Lak (Darlak) Province, ca 15 km of Buon Ma Thuot, Eakmat, 450 m, 1–5.05.1986, col. L.N. Medvedev, 1 spec. (**holotype**, Rc 6358); Dong Nai Province, Ma Da Forest, *Dipterocarpus* area, soil samples, 19.10.1994, col. N.V. Beliaeva, 1 spec. (Rc 6555).

###### Diagnosis.

**Figures 15–20.**
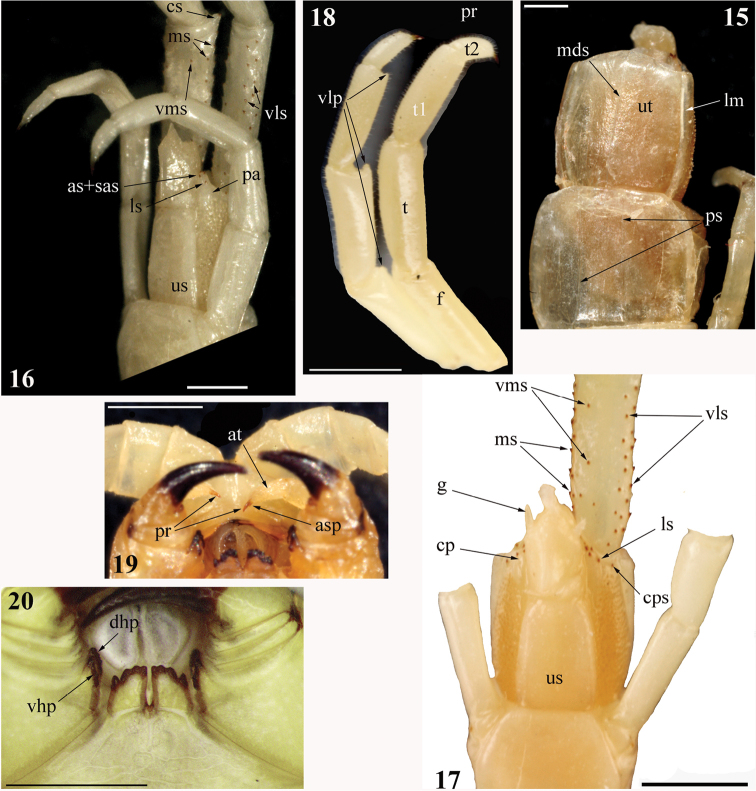
*Tonkinodentuslestes* Schileyko, 1992; Holotype (Rc 6358) **15**LBS 20–21, dorsal view; non-type (Rc 6555) **16**LBS 21 and ultimate prefemora, ventro-lateral view **17**LBS 21 and right ultimate prefemur, ventral view **18** femur, tibia, and tarsus 1 of left (lateral view) and right (medial view) ultimate leg; Holotype (Rc 6358) **19** maxillae 2 and anterior part of forcipular segment, ventral view; *Cormocephalusdentipes* Pocock, 1891, adult Rc 7013 **20** anterior margin of forcipular coxosternite, ventral view; (**as**) – apical spines of coxopleural process, (**asp**) – accessory spines, (**at**) – apical article of telopodite of maxilla 2, (**cp**) – coxopleural process, (**cps**) – coxopleural posterior spine, (**cs**) – corner spine of ultimate prefemur, (**dhp**) – dorsal half of process of trochanteroprefemur, (**f**) – femur, (**g**) – gonopod, (**lm**) – lateral margination, (**ls**) – lateral spine(s) of coxopleural process, (**mds**) – median suture, (**ms**) – medial spine(s) of ultimate prefemur, (**pa**) – coxopleural posterior poreless area, (**pr**) – pretarsus, (**ps**) – paramedian suture(s), (**sas**) – subapical spine(s) of coxopleural process, (**t**) – tibia, (**t1**) – tarsus 1, (**us**) – ultimate sternite, (**ut**) – ultimate tergite, (**vhp**) – ventral half of process of trochanteroprefemur, (**vlp**) – distal ventro-lateral process, (**vls**) – ventrolateral spine(s) of ultimate prefemur, (**vms**) – ventromedial spine(s) of ultimate prefemur.

Cephalic plate lacking any sutures, its posterior margin overlapped by tergite 1; eyes absent (Figs 4, 13). Forcipular tooth-plates well developed and relatively short, with 7 teeth arranged in 2 parallel rows in a chess-board pattern (Fig. 5); trochanteroprefemoral process bisected sagittally (Figs 5, 6). Sternites 2–20 with paramedian sutures (Figs 7, 14). Pleuron with intersclerite membrane clearly visible; spiracles triangular with a 3-part “flap”, slit-like entrance and deep atrium. 21 LBS; the ultimate one visibly shorter than penultimate (Fig. 15). Leg with tarsus 1 considerably longer than tarsus 2, with both tarsal spur and pretarsal accessory spines. Ultimate sternite with poorly developed longitudinal median depression in caudal half. Cylindrical coxopleural process well developed, with spines (Figs 10, 16, 17). Ultimate legs of “common” shape (sensu Schileyko 2009; Figs 8, 12); femur, tibia, and tarsus 1 each with an apically rounded distal ventro-lateral process (Figs 8, 12, 18).

###### Composite redescription.

[data concerning the non-type specimen in square brackets]

Length of body ca 45 [34] mm. Color in ethanol: entire animal uniformly yellow-brownish (Figs 2, 3) [pale yellow, nearly white; Fig. 8]. Body and legs with a very few minute setae.

Antennae of 19 articles (in the both specimens left antenna of 19 and right one of 18, as the corresponding apical article seems to be broken off), reaching the anterior margin of tergite 5 [5.5–6] when reflexed. Basal articles 6 or 7, with a very few long setae, subsequent articles densely pilose. Basal antennal articles flattened.

Cephalic plate (Figs 4, 13) without any sutures, rounded and remarkably narrower than tergite 1; its posterior margin covered by the latter. No light spots at the place of ocelli.

Maxillae 2: the second article of telopodite distally with dorsal spur. Dorsal brush very poorly visible, consisting of short, delicate and transparent setae; apical setae no longer than pretarsus. Uniformly brown pretarsus (Fig. 19) simple (not pectinate) and claw-shaped, as long as 1/3–1/4 of the length of the apical article of telopodite; pretarsus with 2 thin accessory spines.

Forcipular segment: coxosternite with shortly branched medial suture which is as long as 1/3 of coxosternal length; 2 short sutures stretched caudo-laterad from median diastema (Fig. 5) [all coxosternal sutures very hardly visible] in the form of an angle of ca 60° [ca 70°]; chitin-lines short but well developed (Fig. 6). Tooth-plates definitely wider than long [visibly higher than in the holotype]; height of tooth margin increasing medially. Each tooth-plate with 7 teeth, fused to various degrees and arranged in 2 parallel rows in a chess-board pattern (Fig. 5), the lateral tooth is the shortest and the most isolated. Basal sutures of tooth-plates form a nearly straight line. Trochanteroprefemural process well developed, divided sagittally into 2 (dorsal and ventral) halves (Figs 5, 6), each half with 2 or 3 lateral tubercles [dorsal halves of both processes with 3, ventral ones (which are visibly smaller) with 2]; the apical end of this process is considerably higher than corresponding tooth-plate. Tarsungula (Fig. 6) of normal length (left one broken off apically in the holotype), ventrally with 2 blunt ridges.

Tergite 1 without sutures (Figs 4, 13), tergite 2 with visible incomplete paramedian sutures, tergites 3–20 with well-developed and complete ones (Fig. 15), tergite 21 with complete median suture. Tergites 15/16–20 with poorly developed lateral margination posteriorly; only tergite 21 definitely marginate. Tergite 21 nearly as wide as long and not narrowed caudad (Fig. 15); its lateral sides slightly rounded and posterior margin evidently rounded. Tergites lacking any median keel.

Sternites 2–20 (Figs 7, 14) with lateral sides practically parallel; 2–20 with complete paramedian sutures; sternites 6/7–18/19 with a well-developed longitudinal median depression, which is wide and deep [very shallow]. Ultimate sternite long and very narrow (Fig. 11), at least twice as long as wide at base [1.5:1; Fig. 17], very slightly narrowing caudad; its posterior margin practically straight [with strongly rounded corners]. Endosternites not recognizable.

Composition of pleuron (Fig. 9) usual for Scolopendrinae, intersclerite membrane well visible. Elongated spiracles triangular with a typical for this subfamily 3-valved “flap” which covers slit-like entrance in a well-developed atrium (Fig. 9).

Legs (Figs 7, 9) with tarsus 1 considerably longer than tarsus 2, legs 1–18 [1–19] with tarsal spur (legs 19–21 of the holotype are lost). Pretarsus long (approximately as long as ¾ of tarsus 2), legs 1–20 with well-developed accessory spines.

Ultimate LBS visibly shorter than penultimate (Fig. 15). Coxopleuron (excluding coxopleural process) visibly longer than sternite 21 (Figs 11, 16), its coxal part very densely pierced with coxal pores of various size, only this coxopleural process and a narrow posterior area remaining poreless [this posterior area visibly broader than in the holotype]. Short, cylindrical coxopleural process (Figs 10, 16) slightly curved dorsad [definitely curved medially and very slightly dorsad], with 2 apical, 2 subapical, and 2 ventral spines close to its base [with 3 apical, 1 subapical, and 1 lateral spine]; 1 or 2 [1] spines at posterior margin of coxopleuron. Coxopleural process practically reaches the caudal margin of the ultimate tergite. Caudal margin of ultimate pleuron virtually straight and lacking spines; coxopleural surface with scattered minute setae. Gonopods well developed (Figs 11, 17).

[Ultimate legs (Figs 8, 12) ca 7 mm long, relatively slender (width of prefemur ca 0.7 mm), prefemur definitely flattened dorsally, other articles cylindrical. Prefemur, femur and tibia practically of the same length (ca 1.7 mm), tarsus 1 considerably longer than tarsus 2 (Figs 8, 12, 18), the latter twice as long as pretarsus. Ventral surface of prefemur spineless, left prefemur with 23 and right one with 20 small ventrolateral spines, remaining ones (23 on left prefemur, 22 on right) disposed ventromedially, medially and dorsomedially (Figs 12, 16, 17). The spines grouped in very indistinct rows or scattered chaotically; corner spine well developed (Figs 12, 16), with 2 apical spines. No tarsal spur; pretarsus slender, sharply contrasting to much thicker tarsus 2, accessory spines absent. Tarsus 1 and tibia visibly broadened apically; femur, tibia and tarsus 1 each with a characteristic distal process ventro-laterally, the latter short and rounded apically (Fig. 8, 12, 18)].

###### Remarks.

The known material consists of two specimens only, neither of which are in perfect condition. More material is needed to investigate the anatomy (e.g. peristomatic structures, foregut, gizzard).

All differences between the holotype and the second specimen are explicable by the latter being a subadult. The much paler and considerably softer cuticle the second specimen suggests that it is newly moulted. Because of this, some delicate structures (e.g. forcipular sutures, leg spurs) are less evident than in the holotype. The most delicate parts (maxillae, antennae, legs) are somewhat deformed (wrinkled) in the holotype, but in the second specimen, the ventral surfaces of the apical articles of the ultimate legs are deformed (unnaturally concave).

Eight specimens of *Cormocephalusdentipes* Pocock, 1891 (Rc 7518, 7013, 7028, 7231, 7233) from India (Assam and Punjab states), Western Nepal and Indonesia (Sumatra, Medan) demonstrate virtually the same structure of the sagittaly bisected process of the forcipular trochanteroprefemur (Fig. 20). As for the chess-board pattern of the arrangement of the teeth of the forcipular tooth-plates in *Tonkinodentus* (Fig. 5), it is unique among the Scolopendromorpha.

###### Discussion.

The genus *Tonkinodentus* conforms to the Scolopendrinae and differs from members of both the Plutoniumidae and Cryptopidae Kohlrausch, 1881 by: (1) the presence of paired sternal longitudinal sutures (Figs 7, 14) vs single median suture, (2) the slit-like spiracles are covered by a “flap” (a synapomorphy that is unique for Scolopendrinae; Fig. 9), with the longitudinal axis of the spiracle parallel to the such of the body vs open oval spiracles, (3) the well-developed, spinulated coxopleural process (Figs 10, 16) vs its virtual absence, and (4) the ultimate legs of “common” shape (sensu Schileyko 2009; Figs 8, 12) vs enlarged, “pincer-shaped” ones in Plutoniumidae or “pocket knife-shaped” ones in Cryptopidae. *Tonkinodentus* also sharply differs from the typical cryptopids (= *Cryptops* Leach, 1814) by having: (1) well-developed forcipular tooth-plates with strongly chitinized teeth, (2) a forcipular trochanteroprefemur with a well-developed process, (3) sternites without transversal sutures, and (4) prefemur of ultimate legs with numerous spines (Fig. 17).

Summing up, the genus *Tonkinodentus* is morphologically the typical representative of the subfamily Scolopendrinae (and namely of the former tribe Scolopendrini Leach, 1814) and is the most similar to the genus *Scolopendra* L., 1758, but differs readily from the latter by the absence of eyes and the peculiarities of the forcipular segment.

**Figure 21.**
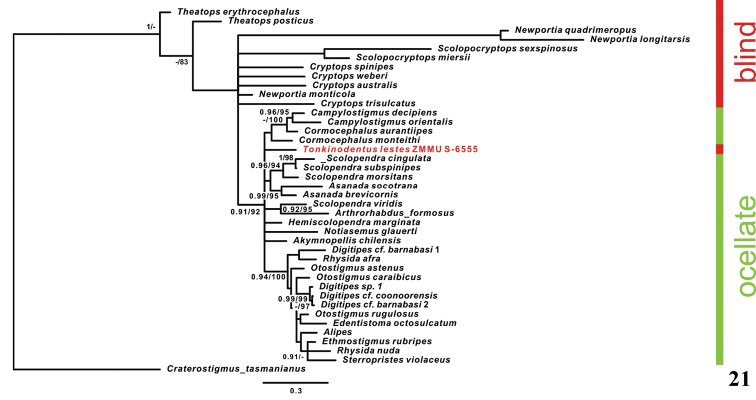
Phylogenetic BI tree reconstructed from alignment of the nuclear gene 28S. (Numbers on tree nodes indicate posterior probabilities (PP > 90) and bootstrap values (BS > 75) for BI/ML, respectively).

**Figure 22.**
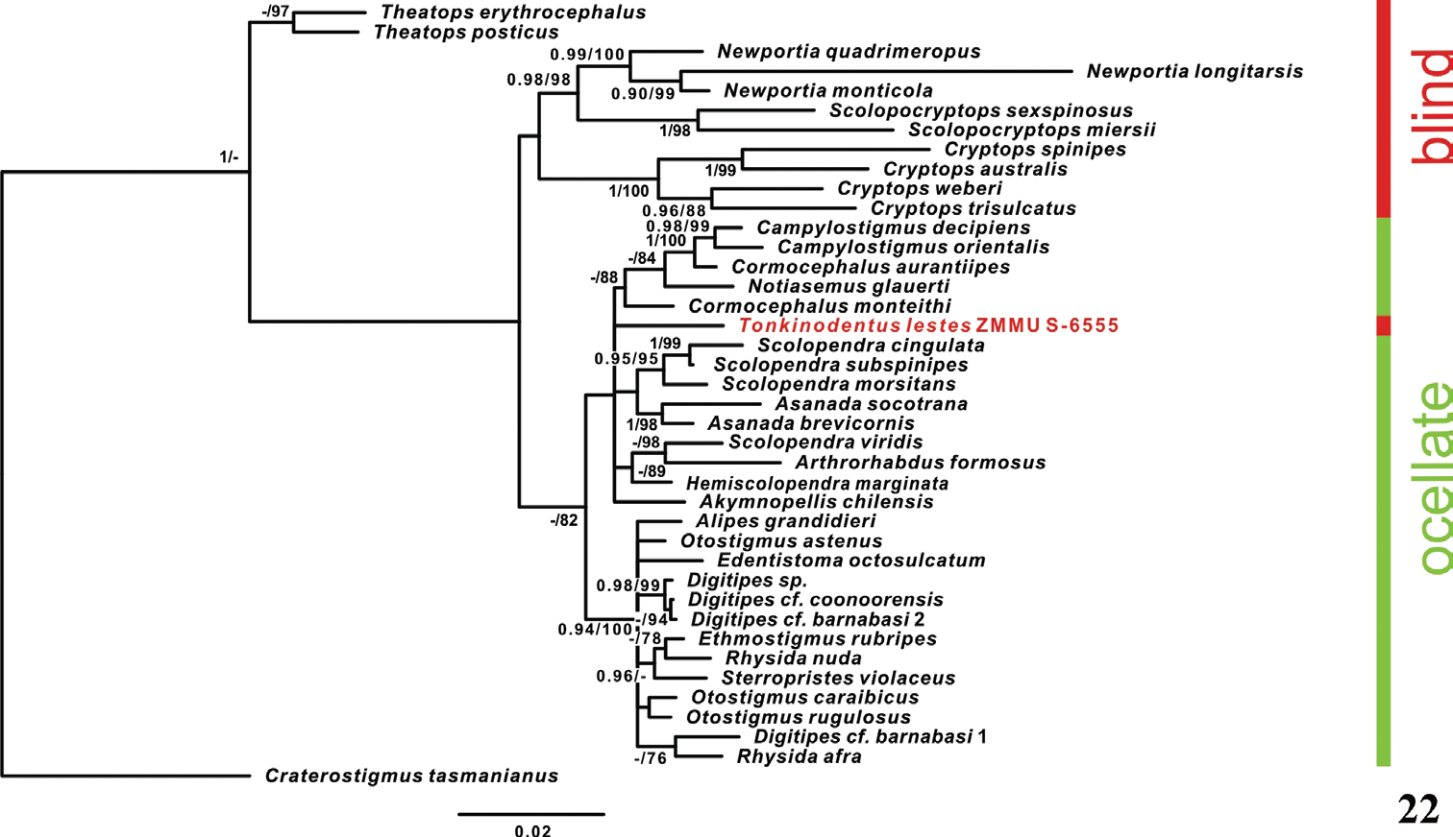
Phylogenetic BI tree reconstructed from concatenated alignment of the nuclear genes 28S + 18S. (Numbers on tree nodes indicate posterior probabilities (PP > 90) and bootstrap values (BS > 75) for BI/ML, respectively).

### 2. Results of the molecular analysis

2.1. Sequence characteristics

We obtained 175 b.p. of 28S rRNA of *Tonkinodentuslestes*. The complete matrix included sequences from 40 species. Information on the length of 28S and 18S fragments and variability is given in Appendix 4 (all data shown for ingroup only). Uncorrected mtDNA genetic distances are given in Appendix 5, 6 (below diagonal).

2.2. Phylogenetic analysis

The results of the phylogenetic analysis are presented in Figures 21 and 22. BI and ML analyses yielded trees that demonstrated essentially similar topologies. Trees based on 28S and 28S + 18S alignments are also rather congruent. Relations of blind species are not resolved in the 28S phylogenetic tree, but non-blind group represents a clade (PP = 0.91, BS = 92). The monophyly of both Scolopendrinae (PP = 0.95, BS = 95) and Otostigminae (PP = 0.94, BS = 100) was supported (Fig. 22). The 28S + 18S phylogenetic tree shows lesser values of support (PP = 0.86, BS = 82) for the non-blind clade, but relations within the blind group are better resolved: two species of *Theatops* Newport, 1844 form a clade (PP = 0.66 (not shown), BS = 97), *Newportia* Gervais, 1847 and *Scolopocryptops* Newport, 1844 form another clade (PP = 0.098, BS = 98), and species of *Cryptops* Leach, 1814 form the third clade (PP = 1, BS = 100). According to both 28S and 28S + 18S topologies, blind *Tonkinodentuslestes* is included in the non-blind clade (= Scolopendridae).

### 3. The taxonomic position of *Tonkinodentus* and the problem of mono- vs paraphyly of the blind scolopendromorphs

The question of the correct taxonomic position of *Tonkinodentus* (in fact, the first eye-less scolopendrid) is connected directly with the problem of mono- vs paraphyly of the blind scolopendromorphs. An origin of the family Cryptopidae sensu Attems (1930), or the “blind clade” sensu Vahtera et al. (2012a), which includes all three eye-less scolopendromorph families (Cryptopidae, Plutoniumidae, and Scolopocryptopidae Pocock, 1896) is a matter of a long discussion. Schileyko (1992) argued the monophyly of the blind scolopendromorphs, stating that the group “Cryptopidae” is not a natural taxon, and tried to support this by producing the first character matrix for the order (Schileyko 1996). This was, however, quite limited and included only 15 genera and eight characters. This viewpoint was, in part, supported by Shelley (1997: 106), who wrote: “… no longer should the present division [of order Scolopendromorpha], based primarily on the presence or absence of eyes, be uncritically accepted”. Shelley (2002: 2) later wrote: “Based on anatomical and biogeographical considerations (discussed by Shelley (1997)), I return the Scolopocryptopinae to full family status from a subfamilial position under the Cryptopidae.”

It is interesting that the results of the purely morphological investigations demonstrate that the para- vs monophyly of the blind scolopendromorphs depends on the parameters of the analysis (Edgecombe and Koch 2008; Edgecombe and Koch 2009; Di et al. 2010) and that “The status of blind Scolopendromorpha as a grade or clade remains an open question” (Edgecombe and Koch 2009: 311). These conclusions have been supported by Koch et al. (2010: 70): “The shortest cladograms include two alternative resolutions of blind scolopendromorphs”.

In contrast, both molecular and/or combined analyses supported the monophyly of the eye-less clade (Edgecombe and Giribet 2004; Vahtera et. al. 2012a). Edgecombe and Giribet (2004: 125) wrote: “The cryptopid clade is present across most of parameter space for combined morphological and molecular data (…), leading us to favor the hypothesis that loss of ocelli in Cryptopidae occurred once and defines a monophyletic group”. Also Vahtera et. al. (2012a, 2013) considered a single loss of ocelli in Scolopendromorpha as the most parsimonious. Confirming these conclusions Bonato et al. (2017: 2) stated that Plutoniumidae, Cryptopidae and Scolopocryptopidae are a “… well-supported monophyletic subgroup, informally labelled as the ‘blind clade’...”.

Morphology and Sanger sequence data reviewed above have been inconclusive with regards to the monophyly of a clade uniting the blind scolopendromorphs (except for *Tonkinodentus*); this grouping is robustly supported by phylogenomic data (Fernández et al. 2016). All of 20 analyses using different gene partitions, optimality criteria (Bayseian Inference or Maximum Likelihood), or tree-inference algorithms recover this group with strong support.

The only mention of *Tonkinodentus* within this discussion has been made by Vahtera et al. (2012a: 14), who wrote: “Our data are lacking the monotypic blind scolopendrid genus *Tonkinodentus* Schileyko, 1992. Morphology supports an assignment of this rare genus to Scolopendridae (Schileyko 2007) but this hypothesis remains yet to be tested in terms of the molecular data. As such, although we postulate a single origin for blindness in three families of Scolopendromorpha, an independent loss of ocelli within Scolopendridae (in *Tonkinodentus*) is probably based on published morphological evidence for the affinities of *Tonkinodentus*”. Summing up, the results of the first molecular approach applied to this peculiar genus should be of the special importance for this discussion.

As it was already noted above, Schileyko (2007) assigned *Tonkinodentus* to the family Scolopendridae (sensu lato), so the precise taxonomic position of *Tonkinodentus* within the family remains indefinite. In the most current general review of scolopendromorph genera, Edgecombe and Bonato (2011: 400) included this genus in the former tribe Scolopendrini Leach, 1814, but provided no arguments for doing so. Later, using a combined morphological and molecular approach, Vahtera et al. (2013: 578) showed that “the tribe Asanadini [Verhoeff, 1907] nests within Scolopendrini for molecular and combined datasets”, thus reducing both tribes, but without formalizing their new statuses. The molecular data confirms that *Tonkinodentus* nests in the family Scolopendridae, or in the subfamily Scolopendrinae (Figs 21, 22), and thus, the discovery of the first eye-less scolopendrid is confirmed.

## Conclusions

Work with ancient DNA from long-preserved museum collections is now an important and developing, but complicated, phylogenetic approach. In this study of *T.lestes*, DNA was so degraded and in so small an amount that two different methods of DNA extraction were used, and only two short fragments of 28S rRNA were obtained.

Both morphological and the first molecular data unequivocally support the position of blind *Tonkinodentus* inside sighted Scolopendridae. The position of *Tonkinodentus* among the members of Scolopendrinae (i.e. non-blind scolopendromorphs with slit-like spiracles covered by a “flap”) is well confirmed by morphological data, but has quite low nodal support in our phylogenetic analysis. More fresh materials are necessary to complete both internal anatomical and molecular studies of this enigmatic scolopendrid.

The position of *T.lestes* within the sighted family Scolopendridae coincides with hypothesis that blind scolopendromorphs are non-monophyletic, although phylogenomics strongly supports monophyly of a clade of the three obligately blind families.

## Supplementary Material

XML Treatment for Tonkinodentus

XML Treatment for Tonkinodentus lestes

## Figures and Tables

**Figure:**
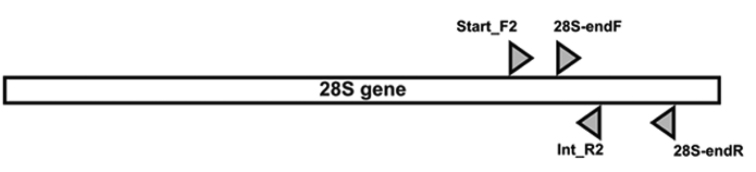
Simplified scheme of the primer positions on 28S gene.

## Appendix 1

**Table:** Primer pairs used in this study.

Fragment	Primer name	Sequence of 3'–5'	Annealing temperature
1	28S-endF	3'-GGAGTCCCGGGAAGAGTTGTC-5'	56 °C
1	28S-endR	3'-TACGGTCCGGCGCGAAAATCA-5'	
2	startF_2	3'-CCGAGCGACCGAAAGGGAATC-5'	58 °C
2	IntR_2	3'-AGTCCGTCCCTTACAAAGAAAAGACAACTCT-5'	

## Appendix 3

**Table:** Sequences used in this study.

species	28S	18S
* Akymnopellis chilensis *	HQ402521	HQ402503
* Alipes grandidieri *	HM453273	KF676422
* Arthrorhabdus formosus *	HQ402522	HQ402504
* Asanada brevicornis *	HQ402523	HQ402505
* Asanada socotrana *	HQ402524	HQ402506
* Campylostigmus decipiens *	HQ402525	HQ402507
* Campylostigmus orientalis *	HQ402526	HQ402508
* Cormocephalus aurantiipes *	HQ402527	HQ402509
* Cormocephalus monteithi *	HM453274	AF173249
* Craterostigmus tasmanianus *	HM453266	AF000774
* Cryptops australis *	AY288708	AY288692
* Cryptops spinipes *	AY288709	AY288693
* Cryptops trisulcatus *	AF000783	AF000775
* Cryptops weberi *	HQ402535	HQ402518
Digitipescf.barnabasi 1	JN003983	–
Digitipescf.barnabasi 2	JN003987	–
Digitipes cf. coonoorensis	JN003979	–
*Digitipes* sp.	JN003980	–
* Edentistoma octosulcatum *	KM492928	KM492930
* Ethmostigmus rubripes *	HM453276	KF676424
* Hemiscolopendra marginata *	HQ402530	HQ402513
* Newportia longitarsis *	HM453281	HM453236
* Newportia monticola *	HQ402531	HQ402514
* Newportia quadrimeropus *	HQ402529	HQ402511
* Notiasemus glauerti *	KF676405	KF676456
* Otostigmus astenus *	HQ402532	HQ402515
* Otostigmus caraibicus *	HQ402533	HQ402516
* Otostigmus rugulosus *	HQ402534	HQ402517
* Rhysida afra *	HQ402536	–
* Rhysida nuda *	HM453277	AF173252
* Scolopendra cingulata *	HM453275	U29493
* Scolopendra morsitans *	HQ402537	HQ402519
* Scolopendra subspinipes *	HQ402538	HQ402520
* Scolopendra viridis *	DQ222134	DQ201419
* Scolopocryptops miersii *	HQ402528	JX422720
* Scolopocryptops sexspinosus *	AY288710	AY288694
* Sterropristes violaceus *	KF676377	KF676428
* Theatops erythrocephalus *	HM453279	AF000776
* Theatops posticus *	HM453280	AY288695
*** Tonkinodentus lestes ***	MK517656	–

## Appendix 4

**Table:** Sequence characteristics. Cons. = conservative sites, Var. = variative sites, Pars.-Inf. = parsimony informative sites.

Locus	Length (b.p.)	Cons.	Var.	Pars.-Inf.	Nucleotide frequencies (%)			
					T/U	C	A	G
**28S**	1743	945	744	381	18.5	25.7	23.8	31.9
**18S**	1913	1662	237	120	23.4	24.7	23.7	28.2

## Appendix 5

**Table 4:** Uncorrected *p*-distances (%) for sequences of 28S nuDNA gene for species (above diagonal). Standard error estimates are shown above the diagonal.

		1	2	3	4	5	6	7	8	9	10	11	12	13	14	15	16	17	18	19	20	21	22	23	24	25	26	27	28	29	30	31	32	33	34	35	36	37	38	39
**1**	*** Akymnopellis chilensis ***		0.83	0.73	0.77	0.76	0.82	0.96	0.87	0.63	2.00	1.82	1.84	0.84	1.83	1.24	1.26	1.53	1.66	0.77	0.76	0.98	0.95	1.15	1.58	0.66	0.66	0.68	0.84	0.60	0.65	1.06	1.88	0.75	1.65	0.66	0.79	0.89	0.96	1.03
**2**	*** Alipes grandidieri ***	8.34		0.83	1.02	0.86	1.04	1.03	1.12	0.73	1.97	2.08	2.04	1.02	1.58	1.01	0.93	1.13	1.46	0.61	1.05	0.99	1.23	1.22	1.68	0.67	0.58	0.66	0.89	0.84	0.78	1.40	2.19	0.99	1.27	0.66	0.67	0.92	1.03	1.03
**3**	*** Arthrorhabdus formosus ***	8.33	10.27		0.78	0.69	0.84	0.91	0.94	0.72	2.04	1.83	1.85	0.82	1.89	1.39	1.33	1.66	1.72	0.79	0.83	1.04	0.95	1.18	1.69	0.71	0.72	0.72	0.84	0.69	0.70	1.07	1.91	0.79	1.64	0.70	0.82	0.87	1.00	1.28
**4**	*** Asanada brevicornis ***	5.07	5.80	6.38		0.69	0.64	0.73	0.73	0.58	n/c	n/c	n/c	0.90	n/c	7.00	n/c	n/c	n/c	0.84	0.66	1.39	0.85	1.14	n/c	0.68	0.73	0.73	0.85	0.60	0.72	0.86	n/c	0.65	n/c	0.74	0.87	1.08	0.89	1.32
**5**	*** Asanada socotrana ***	6.76	7.91	7.64	4.52		0.76	0.80	0.88	0.75	2.26	2.03	2.05	0.76	2.07	1.41	1.46	1.72	1.96	0.82	0.80	1.02	0.95	1.43	1.98	0.68	0.68	0.69	0.79	0.63	0.67	1.07	2.08	0.81	1.92	0.73	0.78	0.86	0.93	1.82
**6**	*** Campylostigmus decipiens ***	5.23	6.00	6.97	3.86	5.30		0.59	0.55	0.61	n/c	n/c	n/c	0.92	n/c	7.00	n/c	n/c	n/c	0.93	0.73	1.45	0.91	1.27	n/c	0.78	0.74	0.75	0.86	0.70	0.79	0.95	n/c	0.71	n/c	0.77	0.96	1.16	1.00	1.67
**7**	*** Campylostigmus orientalis ***	7.04	6.45	8.13	5.10	6.59	3.41		0.74	0.75	n/c	n/c	n/c	0.97	n/c	7.00	n/c	n/c	n/c	1.01	0.77	1.51	0.99	1.27	n/c	0.86	0.85	0.80	0.95	0.75	0.81	0.95	n/c	0.77	n/c	0.80	1.07	1.20	1.09	1.67
**8**	*** Cormocephalus aurantiipes ***	5.14	7.41	8.01	4.97	6.73	2.41	5.04		0.68	n/c	n/c	n/c	0.95	n/c	7.00	n/c	n/c	n/c	1.02	0.77	1.47	0.99	1.27	n/c	0.86	0.88	0.87	0.87	0.65	0.77	0.99	n/c	0.68	n/c	0.87	1.03	1.19	1.10	1.60
**9**	*** Cormocephalus monteithi ***	5.14	7.92	7.40	1.89	6.02	2.07	3.37	2.43		2.11	1.93	1.96	0.88	1.91	1.41	1.39	1.65	1.82	0.69	0.60	0.96	0.90	1.03	1.53	0.67	0.72	0.71	0.71	0.69	0.69	1.22	1.91	0.67	1.72	0.68	0.71	0.78	0.85	1.04
**10**	*** Cryptops australis ***	17.96	19.53	20.76	n/c	19.42	n/c	n/c	n/c	20.00		1.79	1.77	2.26	2.15	2.05	2.03	2.17	2.07	1.97	n/c	2.24	n/c	n/c	2.10	2.00	2.00	2.01	1.98	1.99	2.00	n/c	2.02	n/c	2.02	2.00	1.91	2.04	n/c	n/c
**11**	*** Cryptops spinipes ***	16.27	21.47	19.71	n/c	18.79	n/c	n/c	n/c	17.44	16.94		1.83	2.20	2.25	2.11	2.08	2.24	2.12	1.99	n/c	2.19	n/c	n/c	1.88	1.99	1.91	1.94	1.80	1.86	1.97	n/c	1.89	n/c	1.87	2.02	1.91	1.93	n/c	n/c
**12**	*** Cryptops trisulcatus ***	19.32	21.33	20.33	n/c	19.67	n/c	n/c	n/c	20.45	17.63	18.31		2.20	2.23	2.18	2.14	2.34	2.23	2.07	n/c	2.14	n/c	n/c	1.99	2.05	2.08	2.08	2.04	1.99	1.94	n/c	1.93	n/c	2.10	2.17	1.97	1.93	n/c	n/c
**13**	*** Cryptops weberi ***	11.26	13.36	12.79	7.90	10.80	8.68	9.73	10.13	10.57	21.63	18.37	16.39		2.27	1.66	1.69	2.10	2.26	0.98	0.92	1.03	0.89	1.22	2.28	0.82	0.79	0.79	0.91	0.76	0.82	1.02	2.13	0.94	2.28	0.84	1.01	0.92	0.96	1.65
**14**	**Digitipescf.barnabasi 1**	12.77	8.14	15.81	n/c	13.51	n/c	n/c	n/c	13.98	21.59	23.58	22.87	18.75		1.27	1.23	1.31	1.72	1.57	n/c	2.27	n/c	n/c	2.06	1.53	1.44	1.44	2.08	1.84	1.92	n/c	2.27	n/c	1.72	1.29	1.57	2.02	n/c	n/c
**15**	**Digitipescf.barnabasi 2**	10.58	6.47	14.62	34.04	12.22	34.04	34.04	34.04	13.02	21.63	21.12	22.45	19.66	6.47		0.40	0.00	1.30	0.92	6.81	1.55	6.81	n/c	1.89	0.99	0.97	0.93	1.50	1.42	1.38	3.11	2.19	7.00	1.45	1.09	1.01	1.44	2.39	n/c
**16**	***Digitipes* sp. 1**	10.89	5.10	13.84	n/c	10.79	n/c	n/c	n/c	11.96	21.12	21.23	22.61	18.87	6.41	1.01		0.24	1.36	0.89	n/c	1.55	n/c	n/c	1.90	0.92	0.84	0.84	1.03	0.94	1.44	2.35	1.47	1.39	n/c	1.38	3.52	2.16	1.39	n/c
**17**	** Digitipes cf. coonoorensis **	10.44	5.00	12.99	n/c	12.06	n/c	n/c	n/c	11.40	21.55	20.67	22.12	17.26	6.48	0.00	0.25		1.32	0.99	n/c	1.69	n/c	n/c	2.01	1.06	1.04	0.98	0.97	0.94	1.64	2.00	1.71	1.67	n/c	1.56	11.45	2.21	1.47	n/c
**18**	*** Edentistoma octosulcatum ***	13.53	8.59	14.20	n/c	13.45	n/c	n/c	n/c	14.66	20.65	20.53	22.19	18.86	11.28	6.67	7.16	6.21		1.16	n/c	2.11	n/c	n/c	1.86	1.34	1.41	1.25	1.46	1.44	1.97	n/c	1.75	1.75	n/c	1.71	n/c	2.15	1.38	n/c
**19**	*** Ethmostigmus rubripes ***	8.71	4.61	9.29	4.39	7.71	4.96	6.40	6.19	7.49	20.47	21.47	21.61	12.76	9.65	6.82	5.01	4.21	6.03		0.90	0.95	1.06	1.17	1.71	0.59	0.61	0.55	0.66	0.58	0.87	0.95	0.81	0.78	0.93	0.79	1.30	2.12	1.25	1.17
**20**	*** Hemiscolopendra marginata ***	5.75	6.04	7.74	4.71	5.50	4.70	6.08	5.95	2.06	n/c	n/c	n/c	9.32	n/c	31.91	n/c	n/c	n/c	5.81		1.45	0.95	1.00	n/c	0.84	0.79	0.76	0.91	0.66	0.70	0.92	n/c	0.70	n/c	0.77	1.06	1.13	0.91	0.88
**21**	*** Newportia longitarsis ***	19.24	17.27	20.02	17.20	15.80	17.70	19.25	18.47	15.79	27.74	26.52	25.81	19.45	23.93	20.53	20.11	17.49	26.09	16.61	18.10		1.35	0.53	2.16	1.03	0.98	1.00	1.08	1.01	0.99	1.34	2.25	1.37	2.14	1.03	0.96	0.97	1.20	1.24
**22**	*** Newportia monticola ***	8.42	8.04	11.03	6.50	9.13	7.50	10.11	9.11	4.30	n/c	n/c	n/c	9.10	n/c	31.91	n/c	n/c	n/c	7.24	8.29	15.81		0.53	n/c	1.04	0.96	0.94	1.12	0.93	0.94	0.93	n/c	0.89	n/c	0.95	1.22	1.14	1.03	1.24
**23**	*** Newportia quadrimeropus ***	3.89	4.59	4.24	4.24	6.03	4.95	4.95	4.95	3.18	n/c	n/c	n/c	4.26	n/c	n/c	n/c	n/c	n/c	3.89	3.18	0.71	0.71		n/c	1.20	1.20	1.19	1.47	1.17	1.18	0.97	n/c	1.14	n/c	1.13	1.19	1.16	1.12	1.30
**24**	*** Notiasemus glauerti ***	11.24	13.47	13.39	n/c	17.71	n/c	n/c	n/c	14.00	20.17	20.45	21.51	22.26	15.90	14.46	14.29	15.11	16.29	14.57	n/c	26.93	n/c	n/c		1.70	1.75	1.82	1.63	1.60	1.78	n/c	1.94	n/c	1.76	1.76	1.83	1.83	n/c	n/c
**25**	*** Otostigmus astenus ***	7.09	5.00	10.41	4.73	7.50	5.61	7.22	7.46	7.08	20.41	20.53	21.55	12.55	8.50	6.79	4.94	4.28	7.84	4.63	6.73	18.56	10.66	4.26	13.71		0.56	0.52	0.87	0.71	0.65	1.08	2.06	0.70	1.40	0.55	0.72	0.94	0.97	1.17
**26**	*** Otostigmus caraibicus ***	7.63	4.78	9.20	5.36	8.27	5.75	6.86	7.47	7.30	20.41	20.23	21.27	12.75	9.04	7.03	4.88	5.43	9.32	5.75	6.09	19.57	9.82	4.26	13.39	5.25		0.49	0.85	0.65	0.64	1.06	2.11	0.77	1.32	0.53	0.68	0.88	0.92	1.18
**27**	*** Otostigmus rugulosus ***	7.74	4.95	9.22	5.34	8.12	6.23	6.37	7.37	7.55	20.12	19.94	21.27	12.30	8.75	6.55	5.04	4.44	7.10	4.09	5.87	19.57	8.91	4.24	15.10	4.71	3.87		0.86	0.68	0.65	1.07	2.06	0.73	1.38	0.52	0.64	0.86	1.02	1.17
**28**	*** Scolopendra cingulata ***	8.25	10.99	9.34	4.63	8.05	4.26	5.76	4.80	6.95	19.82	18.16	20.06	13.92	14.33	16.05	15.22	12.88	14.71	9.75	5.18	18.48	6.70	6.41	14.87	10.67	10.50	10.69		0.51	0.90	1.37	2.08	0.86	1.70	0.90	0.86	0.90	1.07	1.87
**29**	*** Scolopendra subspinipes ***	5.57	9.05	7.44	3.22	6.53	3.47	4.49	3.34	5.77	19.64	20.06	20.85	13.06	12.15	14.75	13.51	11.72	13.77	8.55	3.46	18.98	7.18	3.89	13.65	8.33	8.14	8.68	3.25		0.69	1.09	2.07	0.51	1.56	0.68	0.82	0.86	1.01	1.06
**30**	*** Scolopendra viridis ***	7.10	9.75	7.58	5.29	7.21	5.51	6.58	6.46	6.47	19.88	18.81	19.83	12.60	14.68	14.40	12.92	10.91	12.83	9.29	4.69	19.95	8.13	4.24	13.51	8.61	8.35	8.77	9.67	7.68		1.08	1.87	0.69	1.75	0.68	0.78	0.82	1.04	1.13
**31**	*** Scolopocryptops miersii ***	15.59	18.52	15.18	7.69	15.05	7.65	9.43	9.69	14.85	n/c	n/c	n/c	17.69	n/c	46.33	49.70	61.90	n/c	19.07	8.86	25.30	9.58	2.50	n/c	16.53	16.63	15.90	20.96	17.11	16.16		n/c	0.85	n/c	1.05	1.32	1.31	1.34	1.42
**32**	*** Scolopocryptops sexspinosus ***	20.70	25.28	20.46	n/c	18.98	n/c	n/c	n/c	18.90	22.38	20.79	25.14	21.65	25.53	25.82	25.59	25.00	25.21	25.07	n/c	27.16	n/c	n/c	23.33	24.44	24.44	24.44	21.70	21.19	19.48	n/c		n/c	2.11	2.18	2.10	1.86	n/c	n/c
**33**	*** Scolopendra morsitans ***	4.36	6.55	6.44	4.59	5.68	4.34	5.43	3.80	2.63	n/c	n/c	n/c	9.13	n/c	34.04	n/c	n/c	n/c	5.87	4.80	17.25	8.20	4.24	n/c	5.20	6.31	6.42	4.24	2.18	5.00	8.51	n/c		n/c	0.75	0.92	1.08	1.00	1.29
**34**	*** Sterropristes violaceus ***	12.35	6.15	13.83	n/c	13.15	n/c	n/c	n/c	14.24	21.64	21.22	22.70	20.85	10.65	7.56	7.49	7.52	8.84	6.08	n/c	26.32	n/c	n/c	15.06	7.56	6.61	7.44	13.27	11.75	12.54	n/c	25.07	n/c		1.34	1.43	1.86	n/c	n/c
**35**	*** Rhysida afra ***	8.09	6.50	10.41	5.34	8.82	6.13	6.87	7.48	7.98	21.30	20.53	21.61	13.11	6.73	9.62	8.16	4.48	8.26	6.78	6.62	20.31	9.43	3.90	13.51	5.64	6.31	5.46	11.46	9.38	9.22	16.86	24.10	5.81	7.28		0.67	0.91	1.01	1.04
**36**	*** Rhysida nuda ***	8.96	5.15	11.10	4.57	7.14	4.76	7.62	6.84	7.63	19.82	20.53	21.27	13.21	9.68	7.23	5.73	3.54	8.45	4.20	8.09	16.53	11.79	4.24	16.24	6.67	6.16	5.22	10.47	8.42	9.84	18.56	23.08	6.05	7.24	6.48		0.90	0.95	1.17
**37**	*** Theatops erythrocephalus ***	10.68	11.07	11.28	6.75	8.32	6.97	8.40	8.98	8.42	18.21	16.08	20.00	11.77	16.41	15.40	14.52	11.75	15.88	11.56	7.91	15.74	8.01	3.53	16.13	11.43	11.91	11.21	11.36	10.49	11.11	18.53	19.01	7.52	15.99	11.89	11.64		0.82	1.41
**38**	*** Theatops posticus ***	8.63	8.69	9.92	4.81	6.20	5.34	6.23	6.59	7.62	n/c	n/c	n/c	12.45	n/c	20.57	18.88	3.03	n/c	8.91	4.33	17.37	6.00	3.18	n/c	9.87	9.62	10.17	12.50	11.28	10.76	19.09	n/c	5.71	n/c	10.33	8.72	7.44		1.34
**39**	***Tonkinodentuslestes*ZMMU S-6555**	2.29	2.29	3.43	3.43	6.29	5.71	5.71	5.14	2.29	n/c	n/c	n/c	6.32	n/c	n/c	n/c	n/c	n/c	2.86	1.71	2.86	2.86	3.43	n/c	2.86	2.87	2.86	6.94	2.29	2.86	4.00	n/c	3.43	n/c	2.29	2.86	4.00	3.43	

## Appendix 6

**Table:** Uncorrected *p*-distances (%) for sequences of 18S nuDNA gene for species (above diagonal). Standard error estimates are shown above the diagonal.

		1	2	3	4	5	6	7	8	9	10	11	12	13	14	15	16	17	18	19	20	21	22	23	24	25	26	27	28	29	30	31	32	33
**1**	*** Akymnopellis chilensis ***		0.41	0.37	0.32	0.38	0.42	0.30	0.40	0.47	0.42	0.43	0.30	0.29	0.37	0.36	0.28	0.45	0.39	0.37	0.28	0.38	0.36	0.35	0.33	0.29	0.34	0.31	0.30	0.42	0.36	0.35	0.36	0.36
**2**	*** Alipes grandidieri ***	2.67		0.42	0.35	0.41	0.31	0.36	0.45	0.46	0.43	0.45	0.36	0.35	0.28	0.26	0.33	0.42	0.40	0.38	0.32	0.26	0.27	0.26	0.27	0.33	0.37	0.32	0.33	0.42	0.35	0.26	0.36	0.35
**3**	*** Arthrorhabdus formosus ***	2.86	3.01		0.33	0.42	0.32	0.31	0.45	0.49	0.45	0.46	0.32	0.28	0.38	0.33	0.30	0.47	0.40	0.41	0.30	0.35	0.35	0.34	0.35	0.34	0.35	0.31	0.30	0.43	0.37	0.36	0.38	0.37
**4**	*** Asanada brevicornis ***	1.90	1.95	2.23		0.28	0.24	0.24	0.44	0.48	0.44	0.44	0.24	0.21	0.31	0.28	0.21	0.41	0.35	0.37	0.21	0.29	0.28	0.28	0.28	0.19	0.27	0.15	0.26	0.38	0.32	0.28	0.30	0.31
**5**	*** Asanada socotrana ***	2.79	2.61	2.89	1.30		0.31	0.33	0.45	0.53	0.48	0.47	0.34	0.32	0.36	0.34	0.30	0.48	0.39	0.41	0.31	0.34	0.32	0.33	0.33	0.31	0.33	0.27	0.34	0.45	0.39	0.34	0.35	0.35
**6**	*** Campylostigmus decipiens ***	2.33	1.24	1.49	0.74	1.24		0.09	0.46	0.52	0.44	0.44	0.14	0.19	0.30	0.29	0.25	0.33	0.41	0.35	0.20	0.30	0.29	0.28	0.27	0.25	0.31	0.24	0.23	0.36	0.33	0.31	0.38	0.39
**7**	*** Campylostigmus orientalis ***	1.91	2.17	2.08	1.06	1.99	0.08		0.42	0.48	0.42	0.42	0.10	0.18	0.33	0.29	0.20	0.43	0.37	0.38	0.15	0.31	0.30	0.30	0.28	0.24	0.29	0.21	0.23	0.37	0.33	0.30	0.33	0.34
**8**	*** Cryptops australis ***	3.71	3.63	3.72	3.33	3.80	2.32	3.03		0.34	0.32	0.39	0.41	0.40	0.45	0.43	0.38	0.49	0.43	0.42	0.38	0.42	0.42	0.43	0.44	0.41	0.45	0.42	0.42	0.43	0.39	0.43	0.40	0.39
**9**	*** Cryptops spinipes ***	4.49	4.03	4.62	4.13	4.67	3.23	4.03	2.10		0.39	0.40	0.47	0.46	0.49	0.47	0.44	0.53	0.48	0.47	0.45	0.49	0.47	0.48	0.49	0.47	0.48	0.46	0.44	0.48	0.45	0.47	0.43	0.42
**10**	*** Cryptops trisulcatus ***	3.89	3.65	4.02	3.17	3.81	2.41	3.04	1.99	2.72		0.34	0.43	0.44	0.43	0.41	0.40	0.47	0.44	0.42	0.41	0.40	0.40	0.40	0.43	0.44	0.45	0.42	0.41	0.41	0.40	0.41	0.40	0.38
**11**	*** Cryptops weberi ***	3.93	4.02	4.06	3.50	4.09	2.65	3.25	2.65	2.98	1.94		0.43	0.42	0.42	0.43	0.40	0.47	0.46	0.45	0.42	0.43	0.42	0.42	0.43	0.41	0.46	0.41	0.43	0.42	0.42	0.43	0.44	0.45
**12**	*** Cormocephalus aurantiipes ***	1.81	2.02	2.09	1.06	1.99	0.25	0.22	2.99	3.94	3.17	3.38		0.18	0.32	0.30	0.20	0.43	0.37	0.38	0.15	0.31	0.30	0.29	0.29	0.24	0.30	0.21	0.24	0.37	0.33	0.30	0.33	0.34
**13**	*** Cormocephalus monteithi ***	1.69	2.01	1.70	0.89	1.76	0.50	0.65	2.92	3.92	3.15	3.36	0.65		0.32	0.26	0.19	0.42	0.34	0.35	0.15	0.29	0.28	0.27	0.26	0.23	0.28	0.19	0.23	0.38	0.33	0.27	0.31	0.31
**14**	*** Edentistoma octosulcatum ***	2.35	1.30	2.80	1.78	2.39	1.24	2.12	3.30	3.97	3.32	3.58	2.02	1.85		0.23	0.31	0.41	0.38	0.34	0.29	0.22	0.23	0.22	0.22	0.29	0.36	0.29	0.29	0.36	0.33	0.23	0.33	0.33
**15**	*** Ethmostigmus rubripes ***	2.02	1.08	2.25	1.28	2.04	1.16	1.52	3.14	3.92	3.15	3.42	1.53	1.25	0.98		0.25	0.39	0.34	0.32	0.26	0.17	0.13	0.16	0.13	0.29	0.34	0.26	0.27	0.38	0.32	0.12	0.31	0.31
**16**	*** Hemiscolopendra marginata ***	1.75	1.85	1.70	0.84	1.59	0.83	0.82	3.14	4.04	3.10	3.31	0.82	0.71	1.91	1.20		0.41	0.34	0.36	0.18	0.27	0.27	0.26	0.27	0.22	0.26	0.19	0.21	0.38	0.32	0.26	0.30	0.31
**17**	*** Newportia longitarsi ***	3.71	2.78	3.31	2.37	3.10	1.11	2.64	3.28	3.93	3.01	3.49	2.52	2.44	2.79	2.30	2.30		0.19	0.32	0.43	0.39	0.40	0.40	0.40	0.40	0.43	0.38	0.39	0.39	0.34	0.39	0.38	0.36
**18**	*** Newportia monticola ***	3.10	2.53	3.28	2.09	2.65	1.99	2.42	3.48	4.15	3.22	3.87	2.38	2.20	2.70	2.04	2.09	0.56		0.28	0.34	0.36	0.34	0.34	0.36	0.34	0.37	0.31	0.36	0.35	0.28	0.34	0.33	0.30
**19**	*** Newportia quadrimeropus ***	2.77	2.48	3.06	1.98	2.53	1.49	2.26	3.15	3.99	2.88	3.54	2.21	1.98	2.20	1.76	2.04	1.33	1.27		0.36	0.35	0.35	0.35	0.33	0.35	0.36	0.33	0.36	0.34	0.27	0.33	0.31	0.29
**20**	*** Notiasemus glauerti ***	1.64	1.68	1.86	0.83	1.64	0.50	0.49	2.75	3.64	2.87	3.25	0.38	0.54	1.74	1.25	0.60	2.44	2.09	2.04		0.26	0.25	0.24	0.25	0.23	0.28	0.18	0.21	0.37	0.32	0.26	0.29	0.29
**21**	*** Otostigmus astenus ***	2.27	1.05	2.61	1.41	2.10	1.08	1.65	3.13	3.98	3.14	3.52	1.55	1.54	0.94	0.55	1.49	2.44	2.24	2.07	1.27		0.18	0.18	0.19	0.30	0.34	0.27	0.29	0.37	0.34	0.19	0.33	0.33
**22**	*** Otostigmus caraibicus ***	2.02	1.03	2.46	1.39	2.04	1.16	1.63	3.03	3.87	3.04	3.42	1.53	1.36	1.14	0.33	1.30	2.44	2.04	1.93	1.14	0.55		0.16	0.17	0.29	0.35	0.27	0.27	0.38	0.33	0.15	0.33	0.31
**23**	*** Otostigmus rugulosus ***	1.96	1.08	2.35	1.34	1.99	1.08	1.57	3.25	4.09	3.15	3.53	1.47	1.30	0.98	0.49	1.25	2.44	2.09	1.98	1.09	0.61	0.49		0.18	0.27	0.34	0.25	0.27	0.37	0.32	0.17	0.33	0.31
**24**	*** Rhysida nuda ***	1.96	1.25	2.41	1.28	1.93	1.08	1.41	3.14	3.98	3.20	3.42	1.42	1.14	0.92	0.33	1.30	2.51	2.20	1.87	1.14	0.66	0.54	0.60		0.29	0.35	0.25	0.27	0.38	0.33	0.16	0.34	0.32
**25**	*** Scolopendra morsitans ***	1.69	1.85	2.36	0.67	1.76	0.83	1.19	3.19	3.98	3.15	3.36	1.09	0.98	1.74	1.47	1.03	2.23	1.98	1.93	0.98	1.65	1.47	1.41	1.47		0.27	0.17	0.26	0.36	0.32	0.29	0.31	0.30
**26**	*** Scolopendra cingulata ***	2.48	2.52	2.65	1.46	2.35	1.42	1.92	3.72	4.46	3.32	3.78	1.82	1.75	2.47	1.97	1.59	2.53	2.50	2.17	1.64	2.23	2.03	2.03	2.08	1.48		0.23	0.31	0.37	0.33	0.36	0.32	0.35
**27**	*** Scolopendra subspinipes ***	1.69	1.68	1.97	0.45	1.42	0.83	0.98	3.08	3.87	2.93	3.20	0.87	0.76	1.58	1.14	0.71	2.02	1.82	1.76	0.65	1.32	1.14	1.09	1.09	0.54	1.10		0.23	0.35	0.29	0.28	0.28	0.28
**28**	*** Scolopendra viridis ***	1.86	1.85	1.92	1.23	2.05	0.74	1.09	3.19	3.81	3.15	3.42	0.98	0.98	1.74	1.36	0.93	2.23	2.31	2.10	0.76	1.60	1.36	1.41	1.36	1.20	1.76	0.87		0.40	0.32	0.27	0.30	0.31
**29**	*** Scolopocryptops miersii ***	3.50	3.20	3.44	2.53	3.32	1.49	2.76	3.66	4.17	3.18	3.44	2.89	2.71	2.87	2.65	2.71	2.18	2.22	2.06	2.71	2.80	2.71	2.76	2.71	2.48	3.01	2.37	2.76		0.29	0.39	0.34	0.34
**30**	*** Scolopocryptops sexspinosus ***	2.83	2.43	2.90	1.70	2.48	1.33	2.15	3.04	3.88	2.72	3.32	2.11	1.93	2.37	1.99	1.93	1.68	1.55	1.33	1.93	2.24	2.04	2.10	2.04	1.71	2.01	1.60	1.93	1.50		0.31	0.28	0.28
**31**	*** Sterropristes violaceus ***	2.02	1.14	2.52	1.50	2.21	1.24	1.74	3.25	3.92	3.21	3.58	1.64	1.47	0.98	0.33	1.41	2.37	2.09	1.82	1.36	0.66	0.43	0.60	0.60	1.47	2.20	1.25	1.41	2.71	1.93		0.31	0.29
**32**	*** Theatops erythrocephalus ***	2.72	2.27	2.79	1.81	2.54	1.66	2.21	2.99	3.61	2.71	3.55	2.17	1.88	1.99	1.77	1.94	1.96	1.94	1.66	1.93	2.08	1.88	1.99	1.93	1.71	2.18	1.60	1.93	2.34	1.61	1.77		0.20
**33**	*** Theatops posticus ***	2.54	1.98	2.94	1.69	2.30	1.65	2.09	2.87	3.48	2.50	3.31	2.05	1.76	2.09	1.54	1.82	1.96	1.65	1.49	1.71	1.79	1.54	1.60	1.65	1.54	2.28	1.49	1.76	2.22	1.44	1.49	0.83	
